# The History of the Chemo-Free Model in the Treatment of Acute Promyelocytic Leukemia

**DOI:** 10.3389/fonc.2020.592996

**Published:** 2020-11-16

**Authors:** Hong-Hu Zhu

**Affiliations:** Department of Hematology, The First Affiliated Hospital, School of Medicine, Zhejiang University, Hangzhou, China

**Keywords:** acute promyelocytic leukemia, ATRA, ATO, chemotherapy, early death

## Abstract

Acute promyelocytic leukemia (APL) has become a highly curable disease after four decades of endeavors. Thanks to the efforts of investigators throughout the world, the chemo-free concept has become a reality for both low- and high-risk patients. All-trans retinoic acid (ATRA) plus arsenic trioxide (ATO) without chemotherapy has become a first-line treatment for newly diagnosed APL and has been adopted in guidelines or expert recommendations from the NCCN and ELN and in China. Though the regimen has achieved great success, challenges still exist. The rate of early death still has not diminished significantly and is a major obstacle to curing all patients. Leukocytosis is the most important factor for ED, and completely abandoning chemotherapy is dangerous for certain patients in practice. To narrow the gap between guidelines and practice, this review aims to examine the history of the chemo-free model for the treatment of APL in the arsenic-alone era (1974–2002) and the arsenic plus ATRA era (2002–present) and provide practical considerations regarding early death.

## The Evolution of The Chemo-Free Era

### Arsenic Monotherapy

**Figure 1:**
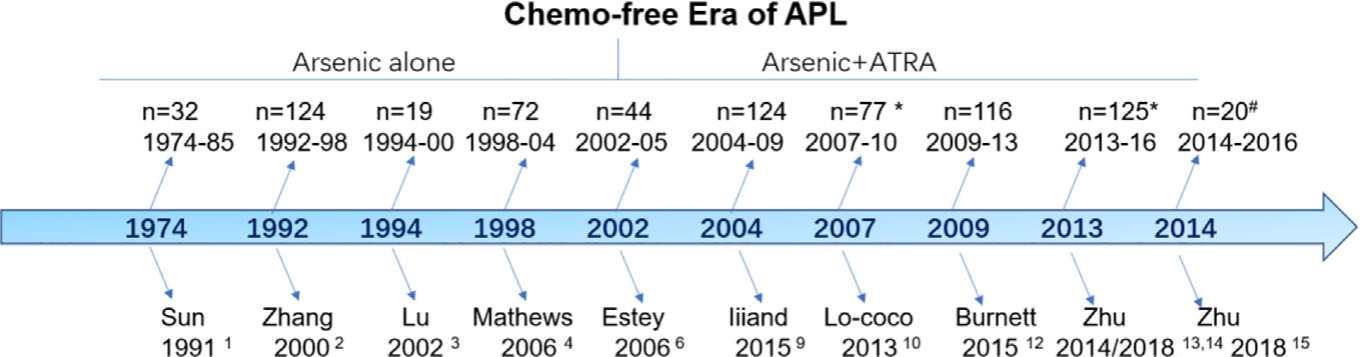
The history of the chemo-free model for newly diagnosed acute promyelocytic leukemia.

Although the term “chemo-free” was introduced for APL in 2011, the history of chemo-free practices can be traced back four decades (**Figure 1**). The chemo-free era can be characterized into two phases: the arsenic monotherapy phase (1974–2002) and the arsenic plus ATRA combination phase (2002–now). Sun et al. from Haerbin, China, reported long-term follow-up results after one injection of ATO-containing monotherapy in 32 patients with newly diagnosed APL between 1974 and 1985 (1). The complete remission (CR) and partial remission rates were 50 and 19%, respectively, and the 5-year overall survival (OS) was 50%. This result was subsequently confirmed by using pure ATO alone in an extension study including 124 patients from the same group (2). Lu et al. first reported the excellent results of a pilot study of 19 patients using oral tetra-arsenic tetra-sulﬁde (As4S4) alone; the authors reported a CR rate of 100% and a 3-year disease-free survival (DFS) of 76.6% (3). However, the total course of arsenic was >3 years in the above studies, which affected the quality of life of the patients. Another two studies from India and Iran shortened the postremission course of ATO to 28 weeks (7 months) and reported similar results (CR rate of 86% and 3-year OS of 86%) (4, 5). The 7-month postremission usage of ATO in the above two protocols provided evidence to design the later arsenic plus ATRA-based chemo-free model.

### ATO Plus ATRA

In 2002, Estey et al. from MD Anderson Cancer Center was the first to investigate the chemo-free model using ATO plus ATRA (CD33-antibody gemtuzumab ozogamicin (GO) for cytoreduction during induction) during induction and postremission treatment (6). Postinduction treatment consisted of four courses of ATO (daily for 5 days/week for 4 weeks every other month; total of 80 doses) and ATRA (2 weeks on/2 weeks off for 7 months). The study included 82 patients, and the CR rate was 92%. The early death rate was 9%, and the estimated 3-year OS was 85%. This result was further confirmed by updated results from the long-term follow-up of the same group, which provided the basis for the subsequent APL0406 study (7, 8). Moreover, IIiand et al. reported an excellent outcome using ATO and ATRA for induction and consolidation, but the inclusion of idarubicin during induction and low-dose cytotoxic agents during maintenance caused this regimen to slightly deviate from the chemo-free goal (9).

Based on the study by Estey et al., Lo-Coco et al. conducted a randomized noninferiority trial, APL0406, using ATRA plus ATO vs. ATRA plus idarubicin for patients with newly diagnosed, non-high-risk (now low-risk) APL (10, 11). The ATRA plus ATO group showed a CR rate of 100% and a 2-year OS of 99% with a median follow-up of 34.4 months. The NCRI AML17 trial aimed to investigate the de-intensification of treatment by randomizing patients irrespective of their risk status between a chemotherapy-free approach (ATO-ATRA) and the ATRA-Chemotherapy (AIDA) regimen (12). ATO was given intravenously at 0·3 mg/kg on days 1–5 of each course, and at 0·25 mg/kg twice weekly in weeks 2–8 of course 1 and weeks 2–4 of courses two to five, the usage of ATO was different from that of the APL0406 study. Gemtuzumab Ozogamicin (GO) was also administered on day1 in the ATO-ATRA arm for high risk patients. CR rate of 94% and 5 year OS was 92% in ATO-ATRA arm.^12^ This study and subsequent update results support ATRA and ATO as first-line treatment not only for low-risk but also for high-risk patients.

### Oral Arsenic Plus ATRA

The next goal was to realize a completely oral, chemo-free model for APL, named the postremission outpatient model. Based on the APL0406 study (10), Zhu et al. first performed a pilot study in 20 low-risk patients using only the oral Realgar-Indigo naturalis formula (RIF) and ATRA without chemotherapy during induction and outpatient-based postremission (13). All patients achieved CR and were still alive and in CR at a median follow-up of 77 months. Subsequently, we demonstrated the noninferiority between oral RIF-ATRA and IV ATO-ATRA in a randomized controlled trial (14). In the end, we extended this concept to high-risk patients, while only incorporating minimal chemotherapy, between April 2014 and September 2016 (15). All 20 patients achieved CR, and the 3-year OS and EFS rates were 100 and 89.4%, respectively (15).

Oral ATO is another arsenic that was first revived by a group from Hong Kong, who thereafter completed a series of clinical trials on this issue (16–18). Recently, Gill et al., in a 15-year prospective follow-up study in 73 patients with relapsed APL, reported 5-year and 10-year OS of 79.5 and 67.3%, respectively (17). Most recently, the same group using oral ATO, ATRA and chemotherapy, reported that both LFS and OS were 100% at 5 years (18). The above studies also inspired interest in the research and development of oral ATO in the USA and Australia. One oral arsenic, named ORH-2014, has completed a phase 1 open-label, dose-escalating study which indicate that ORH-2014 at 15 mg is safe, bioavailable, and provides the required arsenic exposure compared to intravenous ATO at the approved dose (0.15 mg/kg) (19). Moreover, the dose of 10mg is recommended in the future phase 2 and phase 3 trials. Oral ATO developed in Australia is also being evaluated by the ALLG phase I study (APML5) (ACTRN12616001022459).

### Early Death Is the Major Obstacle to Curing All Patients

Early death (ED) is commonly defined as death from any cause within 30 days of diagnosis^12^ or at any time during induction (10, 11). Details about this definition have been systematically reviewed in recent years (19–33). As a result of selection bias, clinical trial data have underestimated the impact of ED, but a series of epidemiologic studies revealed that a signiﬁcant proportion of patients continue to suffer early death (27–29). Encouragingly, however, newer epidemiologic studies now suggest that ED rates may be improving (30–33). According to the US SEER database, ED rates have improved over time (2000–2004, 25.3%; 2005–2009, 20.6%; 2010–2014, 17.1%) in the ATRA plus chemotherapy era (33).

Whether the ED rate can be further reduced in the ATRA plus ATO era remains uncertain. The most important studies of the most representative groups (PETHEMA, GIMEMA, European APL, MRC, etc.) have reported ED rates of around 5% for more than two decades in the ATRA plus chemotherapy era (34–36). Zhu et al. reported that the ED rate in the ATRA plus ATO group was 5.5% (n = 758) based on the data from three large centers in China, which excluded the patients who died without receiving treatment (37). It seems that no difference of ED rate exists between ATRA plus chemotherapy model and ATRA plus arsenic model. Whether ED rate is different between the two models in the population-based study need to be investigated in the future.

### Toxicity of Arsenic and ATRA

The common toxicity of ATO plus ATRA or oral RIF plus ATRA had been systematically reviewed by us recently (38). Liver damage, gastrointestinal toxicity, and headache are common (>10%), while prolongation of the QTc interval and rash are rare (<5%), which is unpredictable before treatment and difficult to perform preemptive therapy. The most important and sometime fatal adverse effect before treatment or during induction therapy with arsenic plus ATRA is leukocytosis, defined as a white blood cell (WBC) count over 10 × 10^9^/L. Lou et al. reported that pretreatment WBCs of 10–50 × 10^9^/L and >50 × 10^9^/L had early death rates of 8.7 and 41.2%, respectively (39). Yoon et al. recently reported that progressive hyperleukocytosis is a relevant predictive marker for differentiation syndrome, early death and subsequent relapse in patients with APL (40). Patients with a WBC before treatment of 10–43 × 10^9^/L that increased to a WBCmax >43 × 10^9^/L experienced an increased risk of early death (33.3%). The multivariate analysis revealed that a WBCmax >43 × 10^9^/L correlated significantly with both early death and differentiation syndrome. Similarly, Therefore, timely minimization of leukocytosis is urgent, and successful prevention of the occurrence of leukocytosis is better.

## Conclusion

The history of APL treatment is almost miraculous. After four decades of endeavors, APL has evolved from a highly fatal disease into a highly curable disease. A chemo-free treatment using only ATRA and ATO in non-high-risk patients was easily applied in clinical practice and is now recommended by current guidelines (41–46). A complete oral and chemo-free model using oral arsenic and ATRA further simplified the procedures and made home-based treatment a reality for more patients.

Apart from ED, the relapse is another major challenge of APL, especially in high-risk patients (27). Until now, no consensus molecular cytogenetic abnormalities at the time of diagnosis can reliably predict the relapse, but monitoring PML-RARA transcripts after treatment is a confidential tool to predict relapse. Currently, ATO plus ATRA is the first choice for the first relapse of APL after front treatment with ATRA plus chemotherapy or ATRA plus ATO. Autologous HSCT remains an appropriate option for younger patients in molecular remission and allogeneic HSCT reserved for patients with persistent molecular positive or with higher degrees of relapse (43).

From the perspective of history, the story of struggling with APL is nearing its end, and this successful model is expected to be attempted on other malignances.

## Author Contributions

The author confirms being the sole contributor of this work and has approved it for publication.

## Conflict of Interest

The author declares that the research was conducted in the absence of any commercial or financial relationships that could be construed as a potential conflict of interest.
